# The predictive validity of the Drinking-Related Cognitions Scale in alcohol-dependent patients under abstinence-oriented treatment

**DOI:** 10.1186/1747-597X-7-17

**Published:** 2012-05-04

**Authors:** Toru Sawayama, Junichi Yoneda, Katsutoshi Tanaka, Norihito Shirakawa, Enami Sawayama, Taichiro Ikeda, Susumu Higuchi, Hitoshi Miyaoka

**Affiliations:** 1 Department of Psychiatry, Kitasato University School of Medicine, 2-1-1 Asamizodai, Minami-ku, Sagamihara, Kanagawa 252-0380, Japan; 2 National Hospital Organization Kurihama Alcoholism Center, 5-3-1 Nobi, Yokosuka, Kanagawa 239-0841, Japan; 3 Department of Occupational Mental Health, Kitasato University Graduate School of Medical Sciences, 1-15-1 Kitasato, Minami-ku, Sagamihara, Kanagawa 252-0374, Japan; 4 Health and Advisory Center for the Well-Being of Spirit and Mind, City of Yokohama, 1735 Toriyama-cho, Kohoku-ku, Yokohama, Kanagawa 222-0035, Japan

**Keywords:** Alcohol-dependent, Treatment outcome, Predictive validity, Drinking-related cognitions scale, Abstinence-oriented treatment, Positive alcohol expectancies, Abstinence self-efficacy, Perception of impaired control, Perception of drinking problems, Denial

## Abstract

**Background:**

Cognitive factors associated with drinking behavior such as positive alcohol expectancies, self-efficacy, perception of impaired control over drinking and perception of drinking problems are considered to have a significant influence on treatment effects and outcome in alcohol-dependent patients. However, the development of a rating scale on lack of perception or denial of drinking problems and impaired control over drinking has not been substantial, even though these are important factors in patients under abstinence-oriented treatment as well as participants in self-help groups such as Alcoholics Anonymous (AA). The Drinking-Related Cognitions Scale (DRCS) is a new self-reported rating scale developed to briefly measure cognitive factors associated with drinking behavior in alcohol-dependent patients under abstinence-oriented treatment, including positive alcohol expectancies, abstinence self-efficacy, perception of impaired control over drinking, and perception of drinking problems. Here, we conducted a prospective cohort study to explore the predictive validity of DRCS.

**Methods:**

Participants in this study were 175 middle-aged and elderly Japanese male patients who met the DSM-IV Diagnostic Criteria for Alcohol Dependence. DRCS scores were recorded before and after the inpatient abstinence-oriented treatment program, and treatment outcome was evaluated one year after discharge.

**Results:**

Of the 175 participants, 30 were not available for follow-up; thus the number of subjects for analysis in this study was 145. When the total DRCS score and subscale scores were compared before and after inpatient treatment, a significant increase was seen for both scores. Both the total DRCS score and each subscale score were significantly related to total abstinence, percentage of abstinent days, and the first drinking occasion during the one-year post-treatment period. Therefore, good treatment outcome was significantly predicted by low positive alcohol expectancies, high abstinence self-efficacy, high perception level of impaired control over drinking, and high perception level of drinking problems measured by DRCS.

**Conclusions:**

The DRCS was considered to have satisfactory predictive validity, which further supports our previous findings. It was suggested that DRCS is a promising rating scale for evaluating multidimensional cognitive factors associated with drinking behavior in alcohol-dependent patients under abstinence-oriented treatment.

## Background

There are various cognitive factors associated with drinking behavior which influence treatment outcome in alcohol-dependent patients [1-6]. For example, lack of perception of drinking problems and impaired control over drinking such as “I don’t have a drinking problem” and “I can control my use of alcohol” is called “denial,” and has long been considered a psychological defense mechanism of alcohol-dependent patients and also a significant obstacle to recovery [5,7]. From the viewpoint of the stages-of-change model by Prochaska and DiClemente [8-10], however, denial as a defense mechanism corresponds to a lack of “readiness to change” or “motivation,” which drives the change in drinking behavior [11,12]. This change model is divided into five stages: precontemplation, contemplation, preparation, action, and maintenance [13,14]. Some alcohol-dependent patients in precontemplation, the first stage of change, can also be viewed as denying their drinking behavior since they are simply unaware of or are underestimating their own drinking problems or impaired control over drinking [15]. It is also known that treatment outcome is generally better in alcohol abusers and alcohol-dependent patients who are at a more progressed stage of change [16-22]. Therefore, the construct including denial, readiness to change and motivation is considered an important cognitive concept upon evaluating “how well alcohol-dependent patients perceive their own drinking problems and impaired control over drinking,” “how determined they are to change their drinking behavior,” etc. [2].

Other important cognitive factors associated with drinking behavior upon determining whether or not alcohol-dependent patients will drink alcohol include outcome expectancies and self-efficacy. Alcohol outcome expectancies include positive expectancies anticipating positive consequences of drinking (such as “I expect to be the life and soul of the party if I have a few drinks”) and negative expectancies anticipating negative consequences of drinking (such as “I expect to have a hangover if I have a few drinks”) [3]. In general, high positive expectancies are considered to bring poor treatment outcome [23,24] and high negative expectancies to bring good treatment outcome [25,26]. Self-efficacy in alcohol abusers and alcohol-dependent patients is defined as the belief held by individuals or the level of self-confidence concerning the ability to resist engaging in drinking behavior [2,6]. In alcohol abusers and alcohol-dependent patients, treatment outcome is considered to be good for patients with high self-efficacy, and poor for patients with low self-efficacy [21,22,27-31].

As described above, cognitive factors associated with drinking behavior in alcohol-dependent patients extend over a wide range and include perception of drinking problems, perception of impaired control over drinking, readiness to change, motivation, alcohol outcome expectancies, and self-efficacy. These cognitive factors are considered to play an important role as predictors of treatment outcome. Therefore, various rating scales have been developed in order to measure cognitive factors associated with drinking behavior (Alcohol Abstinence Self-Efficacy Scale [AASE] [32], Alcohol and Drug Consequences Questionnaire [ADCQ] [33], Alcohol Expectancy Questionnaire [AEQ] [34], Drinking Refusal Self-Efficacy Questionnaire-Revised [DRSEQ-R] [35], Negative Alcohol Expectancy Questionnaire [NAEQ] [36-38], Readiness to Change Questionnaire: Treatment Version [RCQ-TV] [17], Stages of Change Readiness and Treatment Eagerness Scale [SOCRATES] [39], Steps Questionnaire [40], Situational Confidence Questionnaire [SCQ] [41], University of Rhode Island Change Assessment Scale [URICA] [42], etc.) and numerous studies have investigated the relationship between these cognitive factors and treatment outcome. However, the development of a rating scale on lack of perception or denial of drinking problems and impaired control over drinking has not been substantial, even though these are important factors in patients under abstinence-oriented treatment as well as participants in self-help groups such as Alcoholics Anonymous (AA) [2,40,43-46]. In alcohol-dependent patients, cognitive factors associated with drinking behavior are multidimensional and one cognitive factor may not necessarily influence the treatment outcome. A patient may have low self-efficacy or poor perception of impaired control over drinking and, at the same time, have sufficient perception of drinking problems [12].

Therefore, Sawayama et al. [47] developed the Drinking-Related Cognitions Scale (DRCS) for alcohol-dependent patients and investigated its reliability and validity. The DRCS is a 15-item self-reported questionnaire used to briefly evaluate multidimensional cognitive factors associated with drinking behavior in alcohol-dependent patients under abstinence-oriented treatment, including positive alcohol expectancies, abstinence self-efficacy, perception of impaired control over drinking, and perception of drinking problems. Each item is rated on a 6-point scale, where 1 point is given for “Strongly Agree” and 6 points for “Strongly Disagree.” Three out of 15 items are reverse-score items. The DRCS is shown in the appendix (see Additional file 1).

The DRCS is divided into three subscales (expectancy and resignation, perception of impaired control, and perception of drinking problems) with factor analysis. All items have a factor load of 0.40 or greater. The cumulative contribution rate of the three factors was 58.2% (see Additional file 2). “Expectancy and resignation” represents positive alcohol expectancies and abstinence self-efficacy (or resignation concerning abstinence). High scores for “expectancy and resignation” are evaluated as low positive expectancies and high abstinence self-efficacy (or low resignation concerning abstinence). “Perception of impaired control” represents how well one perceives impaired control over drinking (or denial of impaired control). High scores for “perception of impaired control” are evaluated as high perception level of impaired control (or weak denial). “Perception of drinking problems” represents how well one perceives drinking problems (or denial of drinking problems). High scores for “perception of drinking problems” are evaluated as high perception level of drinking problems (or weak denial). Thus, if the total DRCS score is high, cognition associated with drinking behavior is evaluated as adaptive (denial of drinking behavior is weak). Cronbach’s α coefficient in all DRCS items as well as in each subscale was 0.80 or higher, which showed good internal consistency. The value in the analysis of variance intraclass correlation coefficient (ANOVA ICC) with the test-retest method was 0.81 for all items, i.e., sufficiently high and considered to have good reproducibility. Regarding the total DRCS score and each subscale score in the difference of treatment outcome three months after inpatient abstinence-oriented treatment, the scores for the abstinence group were significantly higher compared with the drinking group, which showed good predictive validity.

While a conclusion has not yet been reached regarding whether the treatment goal for alcohol-dependent patients should be “moderation” or “abstinence” [48-53], abstinence-oriented treatment is still commonly provided [53-57]. However, there are few scales available for evaluating lack of perception or denial of drinking problems as well as impaired control over drinking in patients under abstinence-oriented treatment, even those these are important concepts [2,40,43-46]. Therefore, we investigated the relationship between DRCS scores and treatment outcome at the one-year follow-up, in order to further evaluate the predictive validity of DRCS in alcohol-dependent patients under abstinence-oriented treatment.

## Methods

### Participants

The number of participants in this study was 175, including 153 participants from the previous study [47] that surveyed treatment outcome at a 3-month follow-up and an additional 22 people. The participants were voluntarily admitted to the alcohol treatment ward for middle-aged and elderly males at the National Hospital Organization Kurihama Alcoholism Center (Kanagawa, Japan) and all participants met the DSM-IV Diagnostic Criteria for Alcohol Dependence [58].

### Procedure

This study was approved by the Ethics Committee of National Hospital Organization Kurihama Alcoholism Center. Participants received acute-stage treatment (physical treatment as well as treatment of withdrawal symptoms) for three to four weeks at the Internal Medicine Ward, and were transferred to the Alcohol Treatment Ward when they were at a mentally and physically stable stage. After the transfer, the detailed purpose of this study was explained to the participants verbally and in writing, and written consent was obtained from each participant for conducting DRCS as well as cooperating with the outcome survey after discharge.

First, participant characteristics (age, number of admissions to a psychiatry department to treat alcohol dependence, history of alcohol withdrawal symptoms [finger tremor, seizure, and delirium], presence of liver cirrhosis, score on Mini-Mental State Examination [MMSE], status of cohabitation with spouse, employment status, educational history, history of illegal drug usage or abuse, age at first consumption of alcohol, status of alcohol problems in parents or siblings, and score on pre-treatment Self-Rating Depression Scale [SDS]) were assessed prior to implementation of the inpatient treatment program, and DRCS was conducted. After the nine-week inpatient treatment program was completed including assessment and feedback regarding alcohol problems in patients, education on alcohol dependence, motivational approaches to abstinence, coping-skills training, facilitation to participate in a self-help group, etc., DRCS and SDS were conducted again (this inpatient treatment is an abstinence-oriented program). Finally, one year after discharge, an investigation was conducted by face-to-face interview or by phone with patients, families, and municipal welfare office staff. The treatment outcome at the one-year follow-up was assessed by assigning (a) patients with “0” drinking days as the “abstinence group” and (b) patients with “1 or more” drinking days as the “drinking group.”

DRCS was developed to evaluate cognitive factors associated with drinking behavior in alcohol-dependent patients who received abstinence-oriented treatment; therefore, the treatment goal for these patients is total abstinence. However, in order to increase the predictive validity of DRCS, not only total abstinence but also two other treatment outcomes associated with abstinence (the percentage of abstinent days during the one-year post-treatment period and the number of days until the first drinking occasion after discharge) were added as criteria to evaluate the predictive validity. Thus, the predictive validity of DRCS was evaluated by three treatment outcomes during the one-year post-treatment period: (1) total abstinence, (2) percentage of abstinent days and (3) the first drinking occasion.

### Analysis plan

First, the treatment outcome at the one-year follow-up was compared in terms of participant characteristics as well as DRCS scores by chi-square test or unpaired *t*-test. Next, DRCS scores before and after the treatment program were compared by paired *t*-test.

Furthermore, binominal logistic regression analysis was conducted using the forced entry method, with total abstinence as a dependent variable and the total post-treatment DRCS scores, subscale scores, changes in each DRCS score from baseline to end of treatment and age as well as participant characteristics showing significant difference by univariable analysis as independent variables, in order to investigate how the cognitive factors influence treatment outcome under conditions excluding the influence of confounding factors such as participant characteristics.

The extent to which the total post-treatment DRCS scores and subscale scores influence the percentage of abstinent days during the one-year post-treatment period was also investigated. For this purpose, the generalized estimating equation (GEE) analysis was used with the percentage of abstinent days as the dependent variable and the total post-treatment DRCS scores and subscale scores, age, and participant characteristics indicating significant differences in univariable analysis as independent variables.

Finally, the influence of the total post-treatment DRCS scores and subscale scores was examined in terms of the number of days until the first drinking occasion after discharge. Proportional hazard analysis was conducted using the forced entry method, with the first drinking occasion as the dependent variable and the total post-treatment DRCS scores and subscale scores, age, and participant characteristics indicating significant differences in univariable analysis as independent variables.

SPSS 16.0 software was used for statistical analysis.

## Results

### Baseline characteristics of participants

Of the 175 participants in this study, 20 requested discharge during the course of the inpatient treatment program, and 10 were unavailable for follow-up one year after discharge. Thus, the number of subjects for analysis in this study is 145. Among the 145 participants whose answers were successfully obtained in the outcome survey, 53 answers were from face-to-face interviews, 40 from phone interviews, 11 from phone interviews with participants and families, 36 from phone interviews with families only, and five from phone interviews with municipal welfare office staff only.

The demographic characteristics of the participants are shown in Table 1. With regard to patients who had a history of using or abusing illegal drugs, 16 patients used inhalants, three patients used amphetamines, two patients used cannabis, one patient used hallucinogenics, two patients abused sedative-hypnotics and two patients abused analgesics. Five patients had a history of using or abusing two or more types of illegal drugs. There was only one inhalant abuser who was abusing both alcohol and other drugs on admission. No significant difference was found in age, total pre-treatment DRCS scores, and subscale scores between the 145 patients under this study and 30 patients outside of this study (mean age: 49.4 [SD, 7.7] vs. 46.6 [SD, 8.5], unpaired *t*-test = 1.79, df = 173, p = 0.076; mean “expectancy and resignation” score: 28.1 [SD, 6.0] vs. 27.6 [SD, 5.9], unpaired *t*-test = 0.40, df = 173, p = 0.689; mean “perception of impaired control” score: 22.0 [SD, 6.4] vs. 21.7 [SD, 5.5], unpaired *t*-test = 0.27, df = 47, p = 0.787; mean “perception of drinking problems” score: 18.2 [SD, 4.9] vs. 19.0 [SD, 4.4], unpaired *t*-test = −0.80, df = 173, p = 0.425; mean “total” score: 68.3 [SD, 13.7] vs. 68.3 [SD, 10.6], unpaired *t*-test = 0.01, df = 51, p = 0.992).

**Table 1:** Demographic characteristics of participants (n = 145)

Variables	Mean (SD) or %
Age (Mean [SD])	49.4 (7.7)
Number of admissions to a psychiatry department (Mean [SD])	2.0 (1.6)
History of finger tremor due to alcohol withdrawal (% yes)	80.0
History of seizure due to alcohol withdrawal (% yes)	15.9
History of delirium due to alcohol withdrawal (% yes)	22.8
Presence of liver cirrhosis (% yes)	18.6
MMSE (Mean [SD])	27.8 (2.1)
Cohabitation with spouse (% yes)	48.3
Employment status (% working continuously for more than 3 years)	56.6
Educational history (% graduation from high school or higher)	77.2
History of illegal drug usage or abuse (% yes)	13.8
Age at first consumption of alcohol (Mean [SD])	17.5 (3.2)
Alcohol problems in parent or sibling (% yes)	24.1
Pre-treatment SDS score (Mean [SD])	41.1 (7.2)
Post-treatment SDS score (Mean [SD])	38.4 (8.0)
Pre-treatment DRCS score (Mean [SD])	
Expectancy and resignation	28.1 (6.0)
Perception of impaired control	22.0 (6.4)
Perception of drinking problems	18.2 (4.9)
Total	68.3 (13.7)
Post-treatment DRCS score (Mean [SD])	
Expectancy and resignation	31.3 (5.3)
Perception of impaired control	25.0 (5.9)
Perception of drinking problems	20.8 (4.0)
Total	77.1 (12.8)

SD = Standard deviation; MMSE = Mini-Mental State Examination; SDS = Self-Rating Depression Scale.

### Comparison between pre-treatment and post-treatment DRCS scores

When total DRCS scores and subscale scores were compared before and after treatment, a significant increase was seen for both scores (scores for “expectancy and resignation” increased from 28.1 [SD, 6.0] to 31.3 [SD, 5.3], paired *t*-test = −6.53, df = 144, p < 0.001; “perception of impaired control” increased from 22.0 [SD, 6.4] to 25.0 [SD, 5.9], paired *t*-test = −6.10, df = 144, p < 0.001; “perception of drinking problems” increased from 18.2 [SD, 4.9] to 20.8 [SD, 4.0], paired *t*-test = −8.15, df = 144, p < 0.001; and “total” increased from 68.3 [SD, 13.7] to 77.1 [SD, 12.8], paired *t*-test = −8.97, df = 144, p < 0.001).

### Relationship between participant characteristics and total abstinence at one-year follow-up

The relationship between participant characteristics and total abstinence at the one-year follow-up in the univariable analysis is shown in Table 2 (chi-square test or unpaired *t*-test). Characteristics showing a significant difference between the abstinence group and the drinking group included the number of admissions to a psychiatry department to treat alcohol dependence, history of finger tremor due to alcohol withdrawal, employment status, educational history, and history of illegal drug usage or abuse. A significant difference was not seen between the abstinence group and the drinking group regarding the pre-treatment DRCS scores; however, both the total post-treatment DRCS scores and subscale scores were significantly higher in the abstinence group than in the drinking group.

**Table 2:** Relationship between demographic characteristics of participants and total abstinence at one-year follow-up (n = 145)

	Abstinence group (n = 53)	Drinking group (n = 92)	P-value	Statistic
Age (Mean [SD])	50.4 (7.5)	48.9 (7.8)	0.236	t_143_ = -1.19
Number of admissions to a psychiatry department (Mean [SD])	1.5 (1.2)	2.3 (1.7)	0.001	t_135_ = 3.33
History of finger tremor due to alcohol withdrawal			0.020	Chi_1_^2^ = 5.42
No	16 (30.2%)	13 (14.1%)		
Yes	37 (69.8%)	79 (85.9%)		
History of seizure due to alcohol withdrawal			0.256	Chi_1_^2^ = 1.29
No	47 (88.7%)	75 (81.5%)		
Yes	6 (11.3%)	17 (18.5%)		
History of delirium due to alcohol withdrawal			0.980	Chi_1_^2^ < 0.01
No	41 (77.4%)	71 (77.2%)		
Yes	12 (22.6%)	21 (22.8%)		
Presence of liver cirrhosis			0.087	Chi_1_^2^ = 2.94
No	47 (88.7%)	71 (77.2%)		
Yes	6 (11.3%)	21 (22.8%)		
MMSE(Mean [SD])	28.0 (1.7)	27.7 (2.3)	0.403	t_135_ = −0.84
Cohabitation with spouse			0.113	Chi_1_^2^ = 2.51
Yes	32 (60.4%)	43 (46.7%)		
No	21 (39.6%)	49 (53.3%)		
Employment status			0.014	Chi_1_^2^ = 5.98
Working continuously for more than 3 years	37 (69.8%)	45 (48.9%)		
Working continuously for less than 3 years, or unemployed	16 (30.2%)	47 (51.1%)		
Educational history			0.037	Chi_1_^2^ = 4.34
Graduated from high school or higher	46 (86.8%)	66 (71.7%)		
Graduated from junior high school or dropped out of high school	7 (13.2%)	26 (28.3%)		
History of illegal drug usage or abuse			0.008	Chi_1_^2^ = 7.05
No	51 (96.2%)	74 (80.4%)		
Yes	2 (3.8%)	18 (19.6%)		
Age at first consumption of alcohol (Mean [SD])	18.0 (2.9)	17.2 (3.5)	0.153	t_143_ = -1.44
Alcohol problems in parent or sibling			0.934	Chi_1_^2^ = 0.01
No	40 (75.5%)	70 (76.1%)		
Yes	13 (24.5%)	22 (23.9%)		
Pre-treatment SDS score (Mean [SD])	41.2 (6.6)	41.4 (7.6)	0.937	t_143_ = -0.08
Post-treatment SDS score (Mean [SD])	37.9 (7.8)	38.7 (8.1)	0.552	t_143_ = 0.60
Pre-treatment DRCS score (Mean [SD])				
Expectancy and resignation	29.3 (5.4)	27.4 (6.3)	0.057	t_143_ = -1.92
Perception of impaired control	23.0 (6.0)	21.5 (6.6)	0.172	t_143_ = −1.37
Perception of drinking problems	18.5 (4.3)	18.0 (5.2)	0.611	t_143_ =−0.51
Total	70.8 (13.3)	66.9 (13.7)	0.096	t_143_ =-1.67
Post-treatment DRCS score (Mean [SD])				
Expectancy and resignation	33.3 (3.8)	30.2 (5.8)	< 0.001	t_140_ =-3.79
Perception of impaired control	26.5 (4.8)	24.1 (6.3)	0.013	t_133_ =-2.52
Perception of drinking problems	21.7 (3.1)	20.4 (4.4)	0.041	t_137_ =-2.06
Total	81.4 (10.1)	74.7 (13.6)	0.001	t_134_ =-3.37

SD = Standard deviation; MMSE = Mini-Mental State Examination; SDS = Self-Rating Depression Scale.

Statistical tests used are the chi-square test or the unpaired *t*-test.

### Relationship between total abstinence during the one-year post-treatment period and post-treatment DRCS scores in logistic regression analysis

The adjusted odds ratios for total abstinence during the investigation period are shown in Table 3, calculated by binominal logistic regression analysis. Each post-treatment DRCS subscale score had a strong correlation (Spearman’s rank correlation coefficient for “expectancy and resignation” and “perception of impaired control” was 0.60 [P < 0.01], for “expectancy and resignation” and “perception of drinking problems” 0.48 [P < 0.01], and for “perception of impaired control” and “perception of drinking problems” 0.51 [P < 0.01]); therefore, in order to avoid multicollinearity, the total post-treatment DRCS score and three subscale scores were not entered into the model at the same time, but instead, four models were developed and each of these four scores was entered in the respective models to obtain the odds ratio of each score for total abstinence. Each odds ratio was adjusted by age as well as characteristics indicating significant differences in univariable analysis (number of admissions to a psychiatry department to treat alcohol dependence, history of finger tremor due to alcohol withdrawal, employment status, educational history, and history of illegal drug usage or abuse).

**Table 3:** Adjusted odds ratios for total abstinence during the one-year post-treatment period in logistic regression analysis (n = 145)

	Wald chi-square	df	Adjusted OR (95% CI)	P-value
Expectancy and resignation	7.09	1	1.12 (1.03–1.23) ^a^	0.008
Perception of impaired control	6.82	1	1.10 (1.02–1.18) ^b^	0.009
Perception of drinking problems	4.88	1	1.12 (1.01–1.24) ^c^	0.027
Total	8.55	1	1.05 (1.02–1.09) ^d^	0.003

df = degrees of freedom; OR = odds ratio; CI = confidence interval.

Logistic regression analysis was conducted using the forced entry method, with total abstinence as a dependent variable.

^a^ Adjusted for adjustment factors (age, number of admissions to a psychiatry department, history of finger tremor, employment status, educational history, and history of illegal drug usage or abuse). Odds ratio refers to a per-point increment in the score for expectancy and resignation.

^b^ Adjusted for adjustment factors in ^a^. Odds ratio refers to a per-point increment in the score for perception of impaired control.

^c^ Adjusted for adjustment factors in ^a^. Odds ratio refers to a per-point increment in the score for perception of drinking problems.

^d^ Adjusted for adjustment factors in ^a^. Odds ratio refers to a per-point increment in the total score.

Both the total DRCS score and each subscale score were significantly related to total abstinence during the one-year investigation period. The adjusted odds ratio (AOR) for total abstinence by one-point increase of each DRCS score was 1.12 for “expectancy and resignation,” 1.10 for “perception of impaired control,” 1.12 for “perception of drinking problems,” and 1.05 for “total” score.

However, the odds ratio for changes in each DRCS score adjusted under the same conditions was not significant in terms of total abstinence (changes in “expectancy and resignation”: AOR = 1.01, 95% CI [confidence interval] = 0.95–1.08, Wald chi-square = 0.16, df = 1, p = 0.690; changes in “perception of impaired control”: AOR = 1.00, 95% CI = 0.94–1.07, Wald chi-square < 0.01, df = 1, p = 0.988; changes in “perception of drinking problems”: AOR = 1.01, 95% CI = 0.95–1.08, Wald chi-square = 0.16, df = 1, p = 0.690; changes in “total”: AOR = 1.00, 95% CI = 0.97–1.04, Wald chi-square = 0.06, df = 1, p = 0.813).

### Relationship between percentage of abstinent days during the one-year post-treatment period and post-treatment DRCS scores in GEE analysis

The average percentage of abstinent days during the one-year post-treatment period was 45.4% (SD, 34.9) for 92 patients in the drinking group. The total post-treatment DRCS score and subscale scores were significantly related to the percentage of abstinent days in the GEE analysis with the percentage of abstinent days as the dependent variable (Table 4). It was suggested that the percentage of abstinent days significantly increases with the increase in each DRCS score.

**Table 4:** Relationship between percentage of abstinent days during the one-year post-treatment period and post-treatment DRCS scores in GEE analysis (n = 145)

	Regression coefficient (SE)	Wald chi-square	df	95% CI	P-value
Expectancy and resignation	2.07 (0.58) ^a^	12.68	1	0.93–3.20	< 0.001
Perception of impaired control	1.69 (0.51) ^b^	11.12	1	0.70–2.68	0.001
Perception of drinking problems	2.73 (0.73) ^c^	14.09	1	1.31–4.16	< 0.001
Total	0.97 (0.24) ^d^	16.53	1	0.50–1.44	< 0.001

GEE = generalized estimating equation; SE = standard error; df = degrees of freedom; CI = confidence interval.

GEE analysis was conducted with the percentage of abstinent days as a dependent variable.

^a^ Adjusted for adjustment factors (age, number of admissions to a psychiatry department, history of finger tremor, employment status, educational history, and history of illegal drug usage or abuse). Regression coefficient refers to a per-point increment in the score for expectancy and resignation.

^b^ Adjusted for adjustment factors in ^a^. Regression coefficient refers to a per-point increment in the score for perception of impaired control.

^c^ Adjusted for adjustment factors in ^a^. Regression coefficient refers to a per-point increment in the score for perception of drinking problems.

^d^ Adjusted for adjustment factors in ^a^. Regression coefficient refers to a per-point increment in the total score.

### Relationship between first drinking occasion during the one-year post-treatment period and post-treatment DRCS scores in proportional hazards analysis

The average number of days after discharge until the first drinking occasion was 67.0 days (SD, 75.1) for 92 patients in the drinking group. Both the total DRCS score and each subscale score were significantly related to the first drinking occasion (Table 5). The hazard ratio for the first drinking occasion by one-point increase of each DRCS score was 0.95 for “expectancy and resignation,” 0.94 for “perception of impaired control,” 0.92 for “perception of drinking problems,” and 0.97 for “total” score. It was suggested that the first drinking occasion after discharge significantly decreases with the increase in each DRCS score.

**Table 5:** Hazard ratios for first drinking occasion during the one-year post-treatment period in proportional hazards analysis (n = 145)

	Wald chi-square	df	Hazard ratio (95% CI)	P-value
Expectancy and resignation	6.01	1	0.95 (0.92–0.99) ^a^	0.014
Perception of impaired control	11.48	1	0.94 (0.91–0.97) ^b^	0.001
Perception of drinking problems	8.21	1	0.92 (0.88–0.98) ^c^	0.004
Total	11.32	1	0.97 (0.96–0.99) ^d^	0.001

df = degrees of freedom; CI = confidence interval.

Proportional hazards analysis was conducted using the forced entry method, with the first drinking occasion as a dependent variable.

^a^ Adjusted for adjustment factors (age, number of admissions to a psychiatry department, history of finger tremor, employment status, educational history, and history of illegal drug usage or abuse). Hazard ratio refers to a per-point increment in the score for expectancy and resignation.

^b^ Adjusted for adjustment factors in ^a^. Hazard ratio refers to a per-point increment in the score for perception of impaired control.

^c^ Adjusted for adjustment factors in ^a^. Hazard ratio refers to a per-point increment in the score for perception of drinking problems.

^d^ Adjusted for adjustment factors in ^a^. Hazard ratio refers to a per-point increment in the total score.

## Discussion

In this study, we investigated the predictive validity of DRCS in alcohol-dependent patients under abstinence-oriented treatment. Both the total DRCS score and each subscale score were significantly related to total abstinence during the one-year investigation period. Furthermore, both the total DRCS score and each subscale score were significantly related to the percentage of abstinent days as well as the first drinking occasion after discharge. Therefore, good treatment outcome was significantly predicted by low positive alcohol expectancies, high abstinence self-efficacy, high perception level of impaired control over drinking, and high perception level of drinking problems measured by DRCS. This time, we evaluated the predictive validity of DRCS with three treatment outcomes associated with abstinence. As a result, the total DRCS score and each subscale score were significantly related to their respective treatment outcomes. Accordingly, the DRCS was considered to have good predictive validity. This further supports the findings in the previous study [47]. It was suggested that DRCS is a useful rating scale for evaluating multidimensional cognitive factors associated with drinking behavior in alcohol-dependent patients under abstinence-oriented treatment, including positive alcohol expectancies, abstinence self-efficacy, perception of impaired control over drinking, and perception of drinking problems.

Treatment outcome has been successfully predicted by positive alcohol expectancies as well as abstinence self-efficacy in many previous studies [21-24,28]. On the other hand, relatively few studies have examined how well alcohol-dependent patients perceive their own impaired control over drinking as well as their drinking problems and how the perception level influences treatment outcome [2,40,43‐46], even though perceptions of drinking problems and impaired control over drinking are cognitive factors that have received considerable attention over the years in medical care for alcohol dependence. For example, Gilbert [40] found that in the Steps Questionnaire developed to evaluate attitudes and beliefs related to the first three steps in AA’s 12-Step Program, sober days during the three months after discharge were significantly predicted with “powerlessness” as the subscale reflecting Step 1 (We admitted we were powerless over alcohol―that our lives had become unmanageable; examples include “I cannot control my use of alcohol” and “My life has become unmanageable because of alcohol”). “Powerlessness” may be similar to “perception of impaired control” and “perception of drinking problems” as subscales of DRCS. In SOCRATES, the questionnaire designed to evaluate patients’ motivation to change drinking-related behavior [39], Miller and Tonigan [59] found that “recognition” as the subscale reflecting perception of drinking problems (such as “I have serious problems with drinking” and “My drinking is causing a lot of harm”) was significantly related to frequency and intensity of drinking in outpatients at the proximal follow-up (months 4–9) and distal follow-up (months 10–15). These previous studies are consistent with our study, suggesting that perceptions of impaired control and drinking problems are cognitive factors still of concern from the viewpoint of predicting treatment outcome.

It was suggested that DRCS has good predictive validity in alcohol-dependent patients under abstinence-oriented treatment. However, such validity has not been suggested in the case of problem drinkers or alcohol-dependent patients under moderation-oriented treatment. Heather et al. [45] developed the Impaired Control Scale (ICS), a self-reported rating scale to assess the degree of impaired control over drinking in problem drinkers. “Part 2,” one of the three subscales of ICS, measures the frequency of success in controlled drinking in the last six months. High frequency of success measured in “Part 2” was significantly related to good treatment outcome for problem drinkers under moderation-oriented treatment [60]. Unlike the subject’s “perception of impaired control” in DRCS, the actual frequency of success or failure in controlled drinking is measured in “Part 2” of ICS. Frequency of success in controlled drinking is assumed to be actually higher for problem drinkers with mild dependence or low impaired control compared to alcohol-dependent patients [60-62]. Thus, perception of impaired control over drinking is expected to be lower for problem drinkers compared to alcohol-dependent patients. Furthermore, problem drinkers or alcohol abusers under moderation-oriented treatment might have higher self-efficacy for controlled drinking, leading to lower “perception of impaired control” in DRCS. Based on the above, it might be preferable to administer DRCS to alcohol-dependent patients under abstinence-oriented treatment.

The quantity and frequency of drinking prior to treatment was not evaluated in detail in this study. Also, although the participants in this study met the DSM-IV Diagnostic Criteria for Alcohol Dependence, the severity of alcohol dependence was not evaluated. An important future task would be to expand the application of DRCS to the evaluation of mild cases.

This study is an extension to our previous study [47]. We obtained answers to the outcome survey from 41 people other than participants (36 by phone interviews with families only and five by phone interviews with municipal welfare office staff only); therefore the treatment outcome may not have been strictly evaluated. On the other hand, it seems significant that the treatment outcome after one year was successfully evaluated at a high proportion: 145 out of 155 who completed inpatient treatment, by obtaining answers from families and municipal welfare office staff. It is also considered useful that the predictive validity of DRCS was evaluated by three treatment outcomes including total abstinence, percentage of abstinent days and the first drinking occasion.

## Conclusions

In this study, it was suggested that the DRCS has good predictive validity in alcohol-dependent patients under abstinence-oriented treatment. Both the total DRCS score and each subscale score were significantly related to total abstinence, percentage of abstinent days, and the first drinking occasion during the one-year investigation period. Furthermore, when the total DRCS score and subscale scores were compared before and after treatment, a significant increase was seen for both scores. It was suggested that DRCS is a useful rating scale for assessing treatment effects or predicting treatment outcome in alcohol-dependent patients under abstinence-oriented treatment. There are few scales for evaluating lack of perception or denial of drinking problems as well as impaired control over drinking; DRCS would be highly useful since it can briefly assess abstinence self-efficacy, perception of impaired control over drinking and perception of drinking problems in addition to positive alcohol expectancies in alcohol-dependent patients under abstinence-oriented treatment.

## Competing interests

The authors declare that they have no competing interests.

## Authors' contributions

TS, JY, NS, and SH participated in the conceptualization and design of the study. TS, ES, TI, and HM managed the literature searches and summaries of previous related work. TS and KT undertook the statistical analysis, and TS drafted the manuscript. All authors contributed to and approved the final manuscript.

## Supplementary Material

Additional file 1 Drinking-Related Cognitions Scale (DRCS: Translated into English from Japanese). Click here for file

Additional file 2 Factor analysis of DRCS (maximum likelihood method, promax rotation). Click here for file
