# Supplementary material for: The predictive validity of the Drinking-Related Cognitions Scale in alcohol-dependent patients under abstinence-oriented treatment
Source: Subst Abuse Treat Prev Policy. 2012 May 4;7:17. doi: 10.1186/1747-597X-7-17 (PMC3487873; doi:10.1186/1747-597X-7-17)
Supplement: Additional file 1 — Drinking-Related Cognitions Scale (DRCS: Translated into English from Japanese). [file 1747-597X-7-17-S1.doc]

**Appendix 1: Drinking-Related Cognitions Scale (DRCS: Translated into English from Japanese)**

The following statements describe how you might feel about your drinking. How much do you agree (or disagree) with these statements? Please circle the corresponding number.

This questionnaire is not designed to judge “correct or incorrect” answers, so please be honest about what you think. If you are not completely sure about an answer, please circle the number that seems most appropriate and do not leave any blank responses.

Date: Name:

1. I cannot give up drinking as long as I have stress.

| 1 | 2 | 3 | 4 | 5 | 6 |
| --- | --- | --- | --- | --- | --- |
| Strongly Agree | Somewhat Agree | Slightly  Agree | Slightly Disagree | Somewhat Disagree | Strongly Disagree |

1. Even if I have an opportunity to drink again, I will not drink excessively.

| 1 | 2 | 3 | 4 | 5 | 6 |
| --- | --- | --- | --- | --- | --- |
| Strongly Agree | Somewhat Agree | Slightly  Agree | Slightly Disagree | Somewhat Disagree | Strongly Disagree |

1. I have not drunk so much as to cause trouble to my family or people around me.

| 1 | 2 | 3 | 4 | 5 | 6 |
| --- | --- | --- | --- | --- | --- |
| Strongly Agree | Somewhat Agree | Slightly  Agree | Slightly Disagree | Somewhat Disagree | Strongly Disagree |

1. When I get very irritated, I cannot help drinking.

| 1 | 2 | 3 | 4 | 5 | 6 |
| --- | --- | --- | --- | --- | --- |
| Strongly Agree | Somewhat Agree | Slightly  Agree | Slightly Disagree | Somewhat Disagree | Strongly Disagree |

1. Even if I limit my alcohol consumption, I will eventually return to my previous pattern of drinking.^a^

| 1 | 2 | 3 | 4 | 5 | 6 |
| --- | --- | --- | --- | --- | --- |
| Strongly Agree | Somewhat Agree | Slightly  Agree | Slightly Disagree | Somewhat Disagree | Strongly Disagree |

1. Drinking has not interfered with my work or finances.

| 1 | 2 | 3 | 4 | 5 | 6 |
| --- | --- | --- | --- | --- | --- |
| Strongly Agree | Somewhat Agree | Slightly  Agree | Slightly Disagree | Somewhat Disagree | Strongly Disagree |

1. I cannot control my urge to drink.

| 1 | 2 | 3 | 4 | 5 | 6 |
| --- | --- | --- | --- | --- | --- |
| Strongly Agree | Somewhat Agree | Slightly  Agree | Slightly Disagree | Somewhat Disagree | Strongly Disagree |

1. For low-alcohol beverages such as beer, I do not drink excessively.

| 1 | 2 | 3 | 4 | 5 | 6 |
| --- | --- | --- | --- | --- | --- |
| Strongly Agree | Somewhat Agree | Slightly  Agree | Slightly Disagree | Somewhat Disagree | Strongly Disagree |

1. Drinking problems have interfered with my daily life.^a^

| 1 | 2 | 3 | 4 | 5 | 6 |
| --- | --- | --- | --- | --- | --- |
| Strongly Agree | Somewhat Agree | Slightly  Agree | Slightly Disagree | Somewhat Disagree | Strongly Disagree |

1. Alcohol is my source of energy for life.

| 1 | 2 | 3 | 4 | 5 | 6 |
| --- | --- | --- | --- | --- | --- |
| Strongly Agree | Somewhat Agree | Slightly  Agree | Slightly Disagree | Somewhat Disagree | Strongly Disagree |

1. If I try to drink again and in moderation, the odds that I will succeed are high.

| 1 | 2 | 3 | 4 | 5 | 6 |
| --- | --- | --- | --- | --- | --- |
| Strongly Agree | Somewhat Agree | Slightly  Agree | Slightly Disagree | Somewhat Disagree | Strongly Disagree |

1. I have not caused as many problems related to my drinking as people around me say.

| 1 | 2 | 3 | 4 | 5 | 6 |
| --- | --- | --- | --- | --- | --- |
| Strongly Agree | Somewhat Agree | Slightly  Agree | Slightly Disagree | Somewhat Disagree | Strongly Disagree |

1. There are ways other than drinking to relieve my fatigue from work or housework.^a^

| 1 | 2 | 3 | 4 | 5 | 6 |
| --- | --- | --- | --- | --- | --- |
| Strongly Agree | Somewhat Agree | Slightly  Agree | Slightly Disagree | Somewhat Disagree | Strongly Disagree |

1. Now that I have known the harm of alcohol, I will be able to drink in moderation.

| 1 | 2 | 3 | 4 | 5 | 6 |
| --- | --- | --- | --- | --- | --- |
| Strongly Agree | Somewhat Agree | Slightly  Agree | Slightly Disagree | Somewhat Disagree | Strongly Disagree |

1. It is difficult to lead a pleasant life without drinking.

| 1 | 2 | 3 | 4 | 5 | 6 |
| --- | --- | --- | --- | --- | --- |
| Strongly Agree | Somewhat Agree | Slightly  Agree | Slightly Disagree | Somewhat Disagree | Strongly Disagree |

^a^ Reverse-score item.
