# Supplementary material for: The predictive validity of the Drinking-Related Cognitions Scale in alcohol-dependent patients under abstinence-oriented treatment
Source: Subst Abuse Treat Prev Policy. 2012 May 4;7:17. doi: 10.1186/1747-597X-7-17 (PMC3487873; doi:10.1186/1747-597X-7-17)
Supplement: Additional file 2 — Factor analysis of DRCS (maximum likelihood method, promax rotation). [file 1747-597X-7-17-S2.doc]

**Appendix 2: Factor analysis of DRCS (maximum likelihood method, promax rotation)**

| Item (translated into English from Japanese) | | Factor loading | | |
| --- | --- | --- | --- | --- |
|  | | ER | PI | PD |
| Expectancy and resignation (ER) | | | | |
| 1. | I cannot give up drinking as long as I have stress. | ***0.94*** | -0.04 | -0.06 |
| 4. | When I get very irritated, I cannot help drinking. | ***0.89*** | 0.02 | -0.13 |
| 15. | It is difficult to lead a pleasant life without drinking. | ***0.79*** | -0.06 | 0.14 |
| 7. | I cannot control my urge to drink. | ***0.63*** | -0.02 | 0.00 |
| 10. | Alcohol is my source of energy for life. | ***0.58*** | 0.15 | 0.10 |
| 13. | There are ways other than drinking to relieve my fatigue from work or housework.^a^ | ***0.44*** | 0.12 | 0.18 |
|  |
| Perception of impaired control (PI) | | | | |
| 11. | If I try to drink again and in moderation, the odds that I will succeed are high. | -0.07 | ***1.04*** | -0.03 |
|  |
| 8. | For low-alcohol beverages such as beer, I do not drink excessively. | 0.13 | ***0.79*** | -0.07 |
|  |
| 5. | Even if I limit my alcohol consumption, I will eventually return to my previous pattern of drinking.^a^ | -0.10 | ***0.65*** | 0.07 |
|  |
| 14. | Now that I have known the harm of alcohol, I will be able to drink in moderation. | 0.26 | ***0.60*** | 0.01 |
|  |
| 2. | Even if I have an opportunity to drink again, I will not drink excessively. | 0.04 | ***0.43*** | 0.16 |
|  |
| Perception of drinking problems (PD) | | | | |
| 12. | I have not caused as many problems related to my drinking as people around me say. | -0.17 | 0.12 | ***0.88*** |
|  |
| 3. | I have not drunk so much as to cause trouble to my family or people around me. | 0.20 | -0.10 | ***0.72*** |
|  |
| 6. | Drinking has not interfered with my work or finances. | 0.05 | -0.06 | ***0.70*** |
|  |
| 9. | Drinking problems have interfered with my daily life.^a^ | -0.01 | 0.11 | ***0.50*** |
|  |
| Cumulative contribution rate (%) | | 40.20 | 50.94 | 58.20 |
| ^a^ Reverse-score item. | | | | |
